# Antibody-Negative Nivolumab-Induced Autoimmune Encephalitis: A Case Report and Review of Seronegative Presentations

**DOI:** 10.7759/cureus.102834

**Published:** 2026-02-02

**Authors:** Myriam Hamza, Hannah Randolph, Simon Thebault

**Affiliations:** 1 Neurology, McGill University Health Centre (MUHC), Montreal, CAN; 2 Neuroimmunology, Montreal Neurological Institute, Montreal, CAN

**Keywords:** autoimmune encephalitis, immune checkpoint inhibitor, immune-related adverse events, nivolumab, seronegative encephalitis

## Abstract

Immune checkpoint inhibitors (ICIs) have transformed cancer therapy but can provoke immune-related neurological complications, including autoimmune encephalitis (AIE). We report a 70-year-old woman who developed AIE after 11 months of adjuvant nivolumab for melanoma. Serial cerebrospinal fluid (CSF) studies revealed a lymphocytic pleocytosis and elevated protein that improved with corticosteroids, intravenous immunoglobulin (IVIG), and rituximab. Magnetic resonance imaging (MRI) showed evolving bilateral thalamic and periventricular T2/FLAIR (fluid-attenuated inversion recovery) hyperintensities. CSF and serum were negative for paraneoplastic and antineuronal antibodies. Altogether, this case illustrates a late-onset, antibody-negative form of ICI-associated AIE. We also provide a focused review of ICI-associated seronegative AIE presentations, including epidemiology, clinical features, diagnostic approach, management, and prognosis. This report underscores the need for sustained vigilance for AIE throughout the course of ICI therapy and highlights serial CSF cell counts as a useful biomarker for diagnostic support and treatment monitoring.

## Introduction

Immune checkpoint inhibitors (ICIs) have revolutionised the management of multiple malignancies by enhancing anti-tumour immunity. ICIs work by inhibiting the cellular pathways involved in the suppression of T-cells and their role in maintaining self-tolerance, thereby increasing the immune response against cancerous cells [1]. Nivolumab, one such ICI, is a programmed cell death-1 (PD-1) inhibitor and is widely used in the treatment of melanoma, non-small cell lung cancer, and renal cell carcinoma, among others. Despite its broad indications, an undesired outcome is autoimmunity against potentially any organ [2].

Autoimmune encephalitis (AIE) is a rare (<1%) but serious complication of ICI therapy [3,4]. Among all cases of ICI-induced AIE, seronegative presentations - defined by the absence of detectable neuronal autoantibodies by standard panels in serum or cerebrospinal fluid (CSF) - are the most common, with up to approximately 60-94% of cases lacking autoantibody positivity [5,6]. Nivolumab accounts for a majority of the reported cases of ICI-induced AIE, although it is unknown if nivolumab is particularly prone to causing AIE or if this is due to widespread use of the drug [7-9]. The clinical spectrum of AIE includes limbic and non-limbic encephalitis, meningoencephalitis, and other focal or diffuse central nervous system syndromes [6]. Onset typically occurs within weeks to a few months after ICI initiation but rarely may also occur later in treatment [5-7]. The clinical course ranges from mild, fully reversible syndromes to severe, fatal encephalitis [5,6].

In the absence of a positive antibody test, the diagnosis of seronegative ICI-induced encephalitis relies on clinical presentation (per the 2016 Graus criteria), supportive imaging and CSF features, and exclusion of alternative aetiologies [10]. However, the absence of a confirmatory biomarker and the reliance on a suggestive constellation of nonspecific findings may delay diagnosis and treatment, increasing the risk of mortality [2,6].

We present a case of nivolumab-induced seronegative AIE, highlighting the clinical presentation, diagnostic workup, and therapeutic response. This case underscores two novel aspects of nivolumab-induced seronegative AIE: first, the onset of AIE near the one-year completion of treatment with nivolumab, underscoring the need for sustained vigilance beyond the early treatment period. Second, it demonstrates the utility of serial CSF studies as an objective biomarker of treatment response, where clinical assessment and imaging alone may be insufficient.

## Case presentation

**Figure 1:**
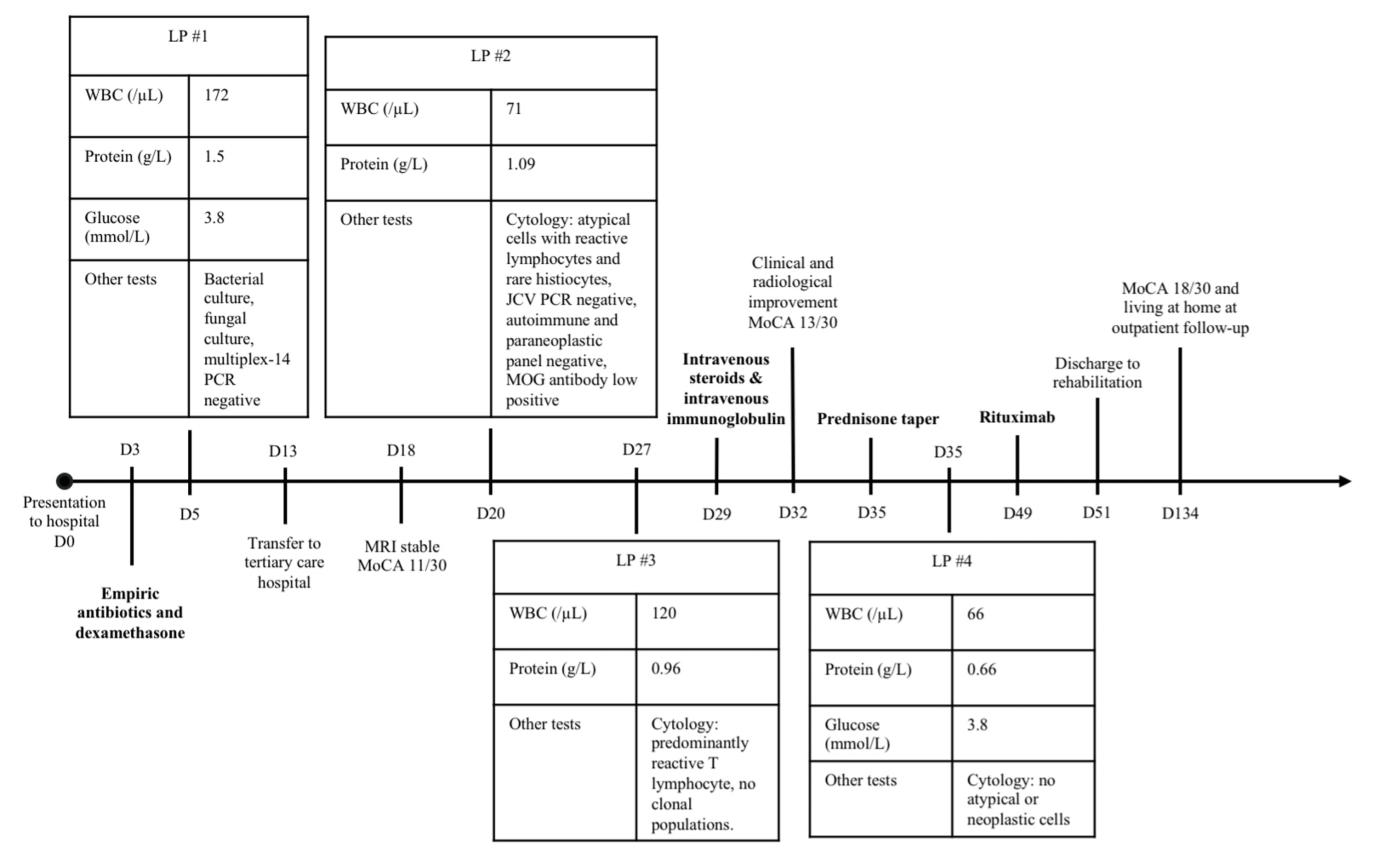
Timeline summarising LP results, treatments provided, and clinical evolution. Infectious agents included in multiplex-14 are *Escherichia coli, Haemophilus influenzae, Listeria monocytogenes, Neisseria meningitidis, Streptococcus agalactiae, Streptococcus pneumoniae*, cytomegalovirus, enterovirus, herpes simplex virus 1, herpes simplex virus 2, human herpesvirus 6, parechovirus, varicella-zoster virus, *Cryptococcus neoformans*, and *Cryptococcus gattii*. Antibodies included in the autoimmune and paraneoplastic panel are GAD65, NMDAR, MPAR, GABABR, CASPR2, AMPAR, LGI1, DPPX, Hu, Yo, Ri, Ma2, CV2, amphiphysin, recoverin, SOX1, titin, Tr/DNER, and Zic4. Abbreviations: D=day; LP=lumbar puncture; WBC=white blood cell; PCR=polymerase chain reaction; JCV=John Cunningham Virus; MOG=myelin oligodendrocyte; MoCA=Montreal Cognitive Assessment.

A previously independent 70-year-old woman presented to a community hospital with a two-week history of progressive disorientation, somnolence, confusion, word-finding difficulty, and gait imbalance (Figure 1). Past medical history included stage III melanoma, resected 14 months prior to presentation, then treated with monthly adjuvant nivolumab starting 11 months prior to presentation, with the last dose administered one month prior to presentation. Her cancer was considered in remission.

**Figure 2:**
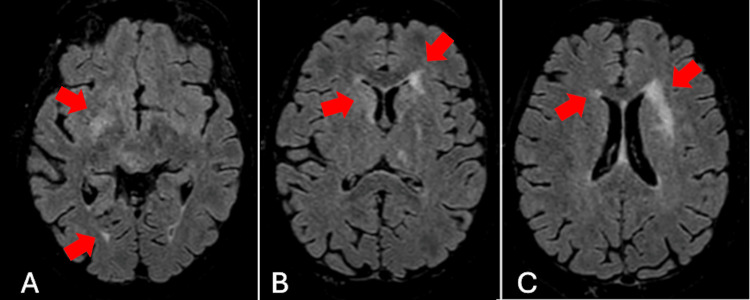
Axial T2/FLAIR MRI demonstrating asymmetrical subcortical (A) and periventricular hyperintensities (B-C) involving the deep white and grey matter at initial presentation, in the context of subacute encephalopathy. Abbreviations: FLAIR=fluid-attenuated inversion recovery; MRI=magnetic resonance imaging

Initial lumbar puncture (LP) revealed a lymphocytic pleocytosis (172 white blood cells (WBC)/µL, 96% lymphocytes; normal=0-10 WBC/µL, 30-90% lymphocytes), elevated protein (1.50 g/L; normal=0.15-0.45 g/L), and normal glucose (3.8 mmol/L; normal=2.5-4.4 mmol/L). The infectious workup was negative for bacterial, viral, and fungal pathogens (see Figure 1 for details of tests performed). Cytology showed reactive T lymphocytes and rare histiocytes consistent with inflammation. Brain magnetic resonance imaging (MRI) showed extensive asymmetric T2/FLAIR (fluid-attenuated inversion recovery) hyperintensities in the bilateral subcortical and periventricular white matter and deep grey matter, with no diffusion restriction or enhancement (Figures 2A-C). Positron emission tomography (PET) excluded malignancy.

She received empiric antibiotics and dexamethasone for possible meningoencephalitis. Antibiotics were discontinued due to negative infectious testing, but dexamethasone was continued on suspicion of an autoimmune process with some improvement in alertness. She was eventually transferred to a tertiary care hospital. The admission exam was remarkable for somnolence, disorientation, mild diffusely increased tone and reflexes, and gait unsteadiness. Her Montreal Cognitive Assessment (MoCA) [11] score was 11/30 with multi-domain deficits. A repeat LP on day 20 after initial presentation, two weeks after having received dexamethasone, demonstrated slightly improved lymphocytic pleocytosis (71 WBC/µL) and persistent elevated protein (1.09 g/L). The repeat MRI was identical. The electroencephalogram (EEG) showed mild diffuse slowing without epileptiform discharges. Infectious workup and autoimmune and paraneoplastic panels in serum and CSF were negative. The myelin oligodendrocyte (MOG) antibody came back low positive, which was deemed nonspecific. A third LP demonstrated worsening pleocytosis (120 WBC/µL) with persistently elevated protein (0.96 g/L). The working diagnosis at this time became nivolumab-induced AIE.

Intravenous (IV) methylprednisolone 1 g daily was given between days 29 and 33, along with IV immunoglobulins (IVIG) 2 g/kg divided over five days between days 30 and 34. A subsequent MRI showed mild radiologic improvement. Her alertness improved, and her MoCA improved slightly to 13/30. LP showed a response (66 WBC/µL, protein 0.66 g/L). This was followed by a slow prednisone taper, with continued gradual clinical improvement. A first dose of rituximab was given on day 49. A follow-up MRI on day 55 showed stability to mild improvement. She was then discharged to her community hospital, followed by inpatient rehabilitation and eventual return home by day 120.

She was seen in follow-up on day 134, where she was noted to have recovered significantly, regaining independence in most activities, while still needing support for more involved tasks. Her MoCA improved to 18/30, with gains in visuospatial, executive, and orientation domains. She remained on a slow prednisone taper with plans to continue rituximab for at least one year. Nivolumab was permanently discontinued.

Written informed consent was obtained from the patient for publication of this case report and any accompanying images.

## Discussion

AIE is an uncommon but serious neurological complication of ICI therapy, particularly relevant in patients treated with PD-1 inhibitors such as nivolumab. Our case demonstrates a prototypical seronegative, nivolumab-induced AIE, distinguished by its late presentation (11 months into therapy) and by the diagnostic and monitoring value of serial CSF studies. Most ICI-associated AIE cases are antibody-negative [6], and nivolumab is the most frequently implicated agent [7,8]; therefore, the absence of neuronal autoantibodies should not delay investigation or treatment, particularly since seronegative AIE often responds well to timely treatment [6,7]. Because clinical presentations are heterogeneous, effective diagnosis relies on maintaining a high index of suspicion, recognising supportive inflammatory CSF findings, and systematically excluding alternative causes.

Based on the FDA Adverse Event Reporting System (FAERS) Public Dashboard, in the USA, nivolumab has been associated with 954 cases of AIE. The first FAERS report of nivolumab-associated AIE appeared in 2014, followed by a sharp rise in reports beginning in 2016, coinciding with the global drug rollout and the expansion of oncologic indications. For comparison, in the USA, there are fewer reported cases of AIE with other ICIs; pembrolizumab, another PD-1 inhibitor, has 795 cases of AIE reported in the FAERS registry since 2014. Ipilimumab, often used in combination with nivolumab, has been associated with 377 cases since 2011. Interpretation of FAERS data is inherently limited by under-reporting, reporting bias, lack of a true exposure denominator, and the inability to establish causality. It therefore remains unclear whether nivolumab confers a truly higher intrinsic risk of AIE after adjusting for drug exposure and indication, or whether this signal primarily reflects its widespread and prolonged clinical use [7,9].

In Table 1, we summarise clinical case reports and case series of nivolumab-associated AIE. One of the most notable features of our case is the late onset, 11 months following nivolumab initiation. Most cases of nivolumab-induced AIE present within the first weeks to months of therapy, with a median onset of 38-55 days [2,4]. For seronegative cases in particular, our review of the literature showed that in five out of the seven seronegative nivolumab-induced AIE cases reported, onset was at or before five weeks after initiation of nivolumab. While delayed presentations were reported, they remained uncommon. Our case underscores that nivolumab-related encephalitis can emerge late in the treatment course, even after near-completion of adjuvant therapy, reinforcing the need for sustained vigilance throughout prolonged or adjuvant nivolumab therapy.

**Table 1:** Review of published seronegative cases of nivolumab-associated autoimmune encephalitis. Abbreviations: FLAIR=fluid-attenuated inversion recovery; MRI=magnetic resonance imaging; CSF=cerebrospinal fluid.

Reference	Tumour type	Onset after nivolumab initiation	Clinical presentation	MRI	CSF	Antibodies tested (returned negative)	Other investigations	Treatment	Outcome
Strik et al., 2017 [12]	Non-Hodgkin lymphoma	32 weeks	Diplopia, dysarthria, ataxia.	Scattered T2/FLAIR hyperintense, contrast-enhancing lesions.	Lymphocytic pleocytosis and elevated protein.	AMPAR, anti-glia, GABAR, myelin-associated antibody, NMDAR, Hu, Ri, Yo, recoverin, SOX1, Zic4, ANA, ANCA.	Biopsy showed T-cell-dominated central nervous system inflammation.	IV corticosteroids, IVIG, cyclophosphamide.	Worsened encephalitis, leading to depression and death by assisted suicide.
Larkin et al., 2017 [4]	Melanoma	Five weeks	Fatigue, aphasia, incoherence, disorientation.	Stable metastases.	Lymphocytic pleocytosis.	Not specified.	None reported.	IV corticosteroids, IVIG, steroid taper.	Resolved encephalitis after 15 days of treatment.
Kawamura et al., 2017 [13]	Lung adenocarcinoma	Four weeks	Dizziness, nausea, nystagmus, dysmetria, ataxia.	Unremarkable.	Pleocytosis, elevated protein.	Not specified.	None reported.	IV corticosteroids, steroid taper.	Improved encephalitis after six days of treatment. Patient died of pneumonia while on a steroid taper.
Schneider et al., 2017 [14]	Non-small cell lung cancer	28 weeks	Somnolence, apathy, aphasia, myoclonus.	Unremarkable.	Pleocytosis, elevated protein and lactate, mildly low glucose.	Amphiphysin, CV2 (CRMP5), Hu, Ri, Yo, Ta/Ma2, Ma1, SOX1, GAD65.	EEG showed moderate background slowing and focal delta slowing and singular sharp waves over the left temporal region.	IV corticosteroids, steroid taper.	Resolved encephalitis after one day of treatment.
Leitinger et al., 2018 [15]	Squamous non-small cell lung cancer	Two and a half weeks	Confusion, anxiety, disorientation, aphasia, seizures, stupor.	Initially normal, then diffuse FLAIR lesions, with subsequent mild improvement.	Lymphomonocytic pleocytosis, elevated protein, normal glucose and lactate.	AMPAR1/2, CASPR2, GABAR, LGI1, NMDAR, amphiphysin, CV2, PNMA2, Ri, Yo, Hu, Tr, recoverin, SOX1, Zic4, titin, GAD65.	EEG showed moderate diffuse slowing.	IV corticosteroids, IVIG.	Worsened encephalitis, leading to comfort care and death.
Cabral et al., 2020 [16]	Non-small cell lung cancer	Four weeks	Aphasia, paraesthesia, amnesia, agitation.	Unremarkable.	Monocytic pleocytosis, elevated protein, normal glucose.	AMPAR, CASPR2, GABAAR, GABABR, GlyR, LGI1, mGluR1, NMDAR, amphiphysin, CV2 (CRMP5), Hu, Ma1, Ta/Ma2, Ri, Yo, SOX1, GAD65.	EEG showed diffuse slowing.	Oral corticosteroids.	Improved encephalitis over two months.
Martínez-Vila et al., 2021 [17]	Melanoma	Three weeks	Amnesia, ataxia, bradypsychia.	Unremarkable.	Lymphomonocytic pleocytosis, elevated protein, normal glucose, positive oligoclonal bands.	Not specified.	EEG showed diffuse slowing.	IV corticosteroids, steroid taper.	Resolved encephalitis after three days of treatment.

Our case presented with a clinical picture of neuropsychiatric decline and confusion. This diffuse pattern of involvement seems to be more common with seronegative ICI-associated AIE cases [6,18], although meningeal involvement and more rhombencephalitic patterns are also reported in seronegative nivolumab-induced AIE specifically. By contrast, seropositive cases, especially those with onconeural antibodies, are more likely to present with focal syndromes (e.g., limbic encephalitis) and are more likely to be associated with underlying neuroendocrine malignancies and other paraneoplastic syndromes [2,6,18].

As in our case, the CSF of patients with nivolumab-induced AIE typically shows a lymphocytic pleocytosis, elevated protein, and preserved glucose on CSF analysis [4,15,16]. This pattern is consistent among the seronegative subgroup (Table 1). In our patient, these abnormalities not only supported the diagnosis but also proved valuable for tracking treatment response. Serial CSF sampling provided a practical objective anchor for inflammatory activity in a setting where clinical change was subtle and difficult to quantify. Modest improvements, such as clearer orientation and small functional gains, were reinforced by parallel biochemical improvement, strengthening confidence in the therapeutic trajectory. This case illustrates how serial CSF cell counts can aid both diagnostic clarification and ongoing monitoring in seronegative ICI-associated AIE.

In our case, MRI demonstrated evolving but slow-to-normalise T2/FLAIR hyperintensities, with radiological improvement lagging behind clear clinical gains. This dissociation between imaging and clinical course is increasingly recognised in ICI-associated AIE [2]. In seronegative cases specifically, our review revealed that the majority of MRIs are normal at initial presentation, despite significant symptoms. In published series, MRI abnormalities are reported in only about half of nivolumab-associated encephalitis cases, most often as nonspecific T2/FLAIR hyperintensities [2]. Some patients with ICI-associated AIE show marked clinical impairment with initially normal imaging, while others, such as our patient, experience meaningful clinical recovery despite persistently abnormal or only subtly improving MRI [6]. Taken together, these observations suggest that while serial MRI remains important for diagnostic confirmation and exclusion of alternative pathology, it may have limited value in monitoring therapeutic response, particularly when the initial MRI is abnormal and clinical improvement is already evident.

Our patient’s clinical course followed the treatment framework commonly used for autoimmune encephalitis: high-dose corticosteroids as first-line therapy with rapid escalation to IVIG when improvement was incomplete, and ultimately rituximab for consolidation therapy [2,4,8,19]. Her early treatment response and steady improvement mirror outcomes described in other nivolumab-induced AIE cases [2,19], as well as the seronegative cases identified in our review. Although the use of rituximab in this context is supported by broader AIE cohorts rather than randomised trial data specific to AIE, pooled survival analyses show a substantial reduction in relapse risk with rituximab, with hazard ratios of approximately 0.29 for first relapse and 0.49 for recurrent relapses [20]. In our case, rituximab consolidation was associated with continued improvement and no relapse of AIE at three-month follow-up. Discontinuation of nivolumab was required in parallel with immunosuppressive therapy, reflecting the necessary balance between oncologic disease control and the management of immune-mediated adverse events.

More broadly, seronegative ICI-associated AIE tends to respond more favourably to immunotherapy than seropositive disease [6,18]. Prompt initiation of corticosteroids and escalation to second-line agents is likely associated with better functional outcomes, while seropositive cases, particularly those with paraneoplastic or intracellular antibodies, often require more aggressive or prolonged immunosuppression and have poorer prognoses [6,7,18]. Up to 88% of seronegative ICI-associated AIE patients achieve good recovery, underscoring the generally favourable trajectory seen in our patient [6]. Our analysis of specifically seronegative nivolumab-induced AIE cases is supportive of this, with the majority of cases improving or resolving after treatment.

## Conclusions

This case illustrates a seronegative ICI-associated AIE with a distinctive late-onset presentation and demonstrates the value of serial CSF studies across diagnosis and treatment. In the absence of neuronal antibodies, evolving CSF pleocytosis and protein elevation provided timely diagnostic support and an objective metric of treatment response when clinical change was subtle. Our review highlights that most ICI-associated AIE cases are seronegative, tend to present with diffuse central nervous system involvement, and generally have a more favourable prognosis than seropositive cases when promptly treated. Notably, our patient developed symptoms 11 months after starting nivolumab, underscoring the need for continued vigilance throughout the full duration of ICI therapy. Together, these observations reinforce the importance of early recognition, careful biochemical monitoring, and rapid escalation of immunotherapy to optimise outcomes.
